# Influence of factor XII deficiency on activated partial thromboplastin time (aPTT) in critically ill patients

**DOI:** 10.1007/s11239-019-01879-w

**Published:** 2019-05-23

**Authors:** Mirjam Bachler, Christian Niederwanger, Tobias Hell, Judith Höfer, Dominic Gerstmeyr, Bettina Schenk, Benedikt Treml, Dietmar Fries

**Affiliations:** 1 Institute for Sports Medicine, Alpine Medicine and Health Tourism, UMIT - University for Health Sciences, Medical Informatics and Technology, Eduard Wallnöfer Zentrum 1, 6060 Hall in Tirol, Austria; 2 Department of Pediatrics, Pediatric Intensive Care Unit, Pediatrics I, Medical University of Innsbruck, Anichstrasse 35, 6020 Innsbruck, Austria; 3 Department of Mathematics, Faculty of Mathematics, Computer Science and Physics, University of Innsbruck, Technikerstraße 13, 6020 Innsbruck, Austria; 4 Department of Anesthesiology and Intensive Care Medicine, AUVA Trauma Centre Salzburg, Academic Teaching Hospital of the Paracelsus Medical University, Dr. Franz Rehrl Platz 5, 5020 Salzburg, Austria; 5 Department of General and Surgical Critical Care Medicine, Medical University Innsbruck, Anichstrasse 35, 6020 Innsbruck, Austria

**Keywords:** Anticoagulation, FXII deficiency, aPTT, Critically ill patients, Thromboprophylaxis

## Abstract

FXII deficiency results in spontaneous prolongation of activated partial thromboplastin time (aPTT), which is widely used to monitor thromboprophylaxis. Misinterpretation of spontaneously prolonged aPTT may result in omission of thromboembolic treatment or even unnecessary transfusion of blood products. This retrospective analysis was performed to calculate a threshold level of FXII resulting in aPTT prolongation. 79 critically ill patients with spontaneous prolongation of aPTT were included. A correlation analysis and a ROC curve for aPTT prolongation predicted by FXII level were created to find the FXII threshold level. Prolongation of aPTT was associated with disease severity. A significant inverse proportionality between FXII and aPTT was seen. A ROC curve for aPTT prolongation, predicted by FXII level (AUC 0.85; CI 0.76–0.93), revealed a FXII threshold level of 42.5%. Of our patients 50.6% experienced a FXII deficiency, in 80.0% of whom we found aPTT to be prolonged without a significantly higher bleeding rate. The FXII deficiency was more common in patients with higher SAPS3 scores, septic shock, transfusion of red blood cells and platelet concentrates as well as in patients receiving renal replacement therapy. Patients with a FXII deficiency and prolonged aPTT less often received anticoagulatory therapy although they were more severely ill. The rate of thromboembolic events was higher in these patients although the difference was not statistically significant. Of all patients with spontaneous aPTT prolongation 50.6% had a FXII level of 42.5% or less. Those patients received insufficient thromboembolic prophylaxis.

## Highlights

The correct setting of anticoagulation especially in critically ill patients is of existential importance. It is mostly based on the aPTT value. Other parameters, such as factor XII, can corrupt this value.

- FXII deficiency is a common finding among critically ill patients with sepsis.
- FXII levels lower than 42.5% frequently leads to an apparent prolongation of aPTT.
- aPTT prolongation due to FXII deficiency may result in omission of thromboprophylaxis.
- With such a complex system as the coagulation can aPTT can not be interpreted without reservation for itself. The search for more independent parameters must be further accelerated.

## Introduction

Pathological standard coagulation parameters are frequently observed in critically ill patients despite the absence of any relevant clinical bleeding symptoms. In addition to increased PT and INR levels and decreased platelet numbers, prolongation of the activated partial thromboplastin time (aPTT) is frequently noticed, especially in this population. In general, increased aPTT is sensitive to decreased levels of Factor VIII, IX, XI and XII as well as to the intake of anticoagulants, anti-phospholipid antibodies and von Willebrand disease [1–3]. Since aPTT measurement is often misused as a predictor for bleeding in critically ill patients [4], the detection of prolongation may lead to insufficient or no antithrombotic therapy or even result in the indiscriminate use of coagulation factors or blood products, especially fresh frozen plasma (FFP), or may even cause urgently required surgical interventions to be postponed [5, 6].

If the aPTT prolongation does not reflect a hypocoagulant state, but is the consequence of confounding factors in the laboratory assay, corrective actions are problematic. Lupus anticoagulants or contact pathway factor deficiencies, such as prekallikrein or FXII deficiency, without any increased risk for bleeding [6–8] may influence the aPTT result.

In critically ill patients with the need for anticoagulant treatment, prolongation of aPTT without any real clinical bleeding tendency is a major problem since aPTT is widely used to monitor the efficacy of anticoagulation [9–11]. Prolongation of aPTT may lead to inadequate thromboprophylactic drug dosing, or in the worst case to no anticoagulation therapy at all [12] and to exposure to allogenic fresh frozen plasma (FFP) transfusion [13]. FFP transfusions and other countermeasures [14] may cause harm if not indicated accurately [15, 16].

Especially in critically ill patients the overall incidence of thromboembolic events (TE) is about 32% [17]. If antithrombotic therapy is withheld, the rate of deep vein thrombosis (DVT) can increase to up to 60% in trauma patients and even to 80% if an acute spinal cord injury is involved [18]. Therefore, correct interpretation of the aPTT is crucial, especially in the critically ill patient population.

aPTT prolongation with the absence of any clinically relevant bleeding symptoms is a common finding in intensive care units. Correct interpretation is difficult, the consequence of which should not be inadequate activities [19]. Even though aPTT prolongation cannot be ascribed solely to FXII deficiency, we chose this factor because of its use as a surrogate marker for contact pathway activation.

Although it has long been known that critically ill patients have increased contact activation with concomitant FXII consumption leading to aPTT prolongation, hardly any studies have dealt with this subject to date.

Since in critically ill patients based on the aPTT time, the current coagulation situation could not be interpreted correctly and thus a wrong treatment decision regarding anticoagulation could be made, we would like to point out in this study that aPTT could be incorrectly prolonged by influencing factors such as FXII deficiency. Therefore, the purpose of this study was to investigate the impact of FXII deficiency on a prolongation of aPTT and thus increase the awareness of treating physicians about the interpretation of aPTT.

## Methods

Primary endpoint of this study was to evaluate whether the FXII levels in patients with and without prolongation of the targeted aPTT range differ and also, as secondary endpoint, to determine a FXII threshold level where aPTT becomes prolonged by a FXII deficiency.

### Inclusion of patients

This retrospective analysis includes clinical data and routine laboratory parameters of 79 critically ill post-surgical patients as well as trauma patients at the General and Surgical Intensive Care Unit of Innsbruck Medical University Hospital.

All medical files of patients with available FXII measurement for whatever reason (e.g. participants in a clinical trial with measurement of various coagulation factors) between 2012 and 2015 were reviewed. A total of 94 patients were found, ten of whom had to be excluded from analysis since they had confounding influences on the coagulation Factor XII assay, such as Lupus anticoagulants, or the administration of direct thrombin inhibitor. Five patients in whom it was unclear whether aPTT was prolonged or not, were excluded. A total of 79 patients were suitable for analysis. For five of these 79 patients limited information was available.

The study protocol was approved by the institutional review board of the Medical University of Innsbruck (EK-No. 1151/2017).

### Data collection

We collected demographic variables such as age, sex, BMI and the reason for ICU admission. Septic shock was defined as the need for catecholamines. As ICU interventions, extracorporeal circuit treatments and anticoagulation therapy were recorded. aPTT prolongation was defined as assay results above the upper norm value of more than 37 s in patients not receiving any anticoagulatory medication. A target aPTT of 60 s was standard in heparin therapy. An overshoot of the targeted upper aPTT range (> 60 s) was defined as aPTT prolongation. If a patient had more than one FXII measurement during the entire ICU stay, the time point of the lowest FXII level was chosen for analysis. At such time other available routine parameters such as fibrinogen, platelets, antithrombin, PT, INR, hemoglobin, hematocrit, leukocytes, C-reactive protein, procalcitonin were collected. The number of transfusions received before FXII measurement was recorded and analysed as the total number of transfusion units and only from 3 days before in order to evaluate the short-term effect of transfusion on the FXII levels. We looked out for any diagnosed thromboembolic event and bleeding complication during the ICU stay.

### Statistical analysis

A mathematician (TH) not involved in data acquisition performed the statistical analyses using R, version 3.4.1. All statistical assessments were two-sided and a significance level of 5% was used. We investigate the relationship between FXII and aPTT using regression analysis and perform ROC curve analysis for an aPTT prolongation predicted by FXII levels; we provide an optimal threshold for FXII as the value corresponding to the point closest to the top-left on the ROC curve. Because the hypothesis of normal distribution was not reasonable for most continuous variables (Shapiro–Wilk normality test) and sample size was not accordingly high, we applied the Wilcoxon rank sum test and Fisher’s exact test to assess differences between patients with and without prolonged aPTT as well as between patients with FXII levels above and those with FXII levels below the threshold. We present continuous data as median (IQR) and categorical variables as frequencies (%) and show effect size and precision with estimated differences between groups for continuous data and odds ratios (OR) for binary variables, with 95% CIs.

## Results

### Patient characteristics

Eligible for final analysis were 79 patients; median age was 64 years, 26.6% of the patients were female. The main affected organ system, responsible for ICU admission, was the cardiovascular system (39.2%) followed by septic complications (17.7%) as seen in Table 1.

**Table 1:** Characteristics of patients stratified for aPTT not prolonged and aPTT prolonged

Characteristic^a^	Total (n = 79)	aPTT not prolonged (= 38)	aPTT prolonged (n = 41)	Estimate with 95% CI^b^	p value^c^
Female gender	21/79 (26.6)	8/38 (21.1)	13/41 (31.7)	1.73 (0.56–5.6)	0.3182
Age (years)	64 (54–73)	58.5 (48.25–69.5)	68 (59–76)	− 8 (− 14 to − 1)	0.0197
BMI (kg/m^2^)	26.55 (22.87–28.7)	26.81 (24.78–28.8)	25.6 (21.18–28.4)	1.9 (− 0.5 to 4.5)	0.115
Reason for ICU admission^d^					
Trauma	8/79 (10.1)	6/38 (15.8)	2/41 (4.9)	0.28 (0.03–1.69)	0.1449
Sepsis	14/79 (17.7)	2/38 (5.3)	12/41 (29.3)	7.28 (1.45–71.97)	0.007
MODS	8/79 (10.1)	2/38 (5.3)	6/41 (14.6)	3.04 (0.5–32.83)	0.266
Cardiovascular system^e^	31/79 (39.2)	16/38 (42.1)	15/41 (36.6)	0.8 (0.29–2.16)	0.6508
Respiratory insufficiency	7/79 (8.9)	2/38 (5.3)	5/41 (12.2)	2.47 (0.37–27.57)	0.4338
Renal failure	9/79 (11.4)	3/38 (7.9)	6/41 (14.6)	1.98 (0.39 to 13.23)	0.4842
Hepatic dysfunction	1/79 (1.3)	0/38 (0)	1/41 (2.4)	Inf (0.02 to Inf)	1
Intestinal tract complication	12/79 (15.2)	6/38 (15.8)	6/41 (14.6)	0.92 (0.22–3.81)	1
Thromboembolic event	5/79 (6.3)	4/38 (10.5)	1/41 (2.4)	0.22 (0–2.32)	0.1898
Scores					
SAPS3 (pts)	71 (54.5–80.5)	59.5 (47.25–71)	78 (72–87)	− 20 (− 29 to − 12)	< 0.0001
SOFA (pts)	12 (8.5–14)	10 (7–12.25)	14 (11–16)	− 4 (− 6 to − 2)	0.0006
Sepsis^d^					
Proven pathogen	59/79 (74.7)	26/38 (68.4)	33/41 (80.5)	1.89 (0.61–6.19)	0.3012
Gram+	40/79 (50.6)	19/38 (50)	21/41 (51.2)	1.05 (0.4–2.78)	1
Gram−	44/79 (55.7)	19/38 (50)	25/41 (61)	1.55 (0.58–4.2)	0.3702
Fungi	38/79 (48.1)	14/38 (36.8)	24/41 (58.5)	2.39 (0.89–6.62)	0.0722
Viral	10/79 (12.7)	5/38 (13.2)	5/41 (12.2)	0.92 (0.19–4.38)	1
Septic shock	26/74 (35.1)	5/35 (14.3)	21/39 (53.8)	6.81 (2.03–27.29)	0.0005
Interventions at lowest FXII level					
ECMO	7/79 (8.9)	4/38 (10.5)	3/41 (7.3)	0.67 (0.09–4.3)	0.7053
RRT	35/79 (44.3)	10/38 (26.3)	25/41 (61)	4.29 (1.53–12.82)	0.003
Anticoagulation therapy	51/74 (68.9)	34/35 (97.1)	17/39 (43.6)	0.02 (0–0.17)	< 0.0001
Length of ICU stay (days)	16 (8–26)	13 (9.25–24)	17 (5–26)	− 1 (− 8 to 5)	0.7871

^a^Binary data are presented as no./total no. (%), continuous data as medians (25th–75th percentile)

^b^Estimated odds ratio for binary and median difference for continuous variables

^c^Differences in groups assessed with Fisher’s Exact Test for binary variables and Wilcoxon Rank Sum Test for continuous variables

^d^Multiple selection possible

^e^Includes post-surgical care or complications

Prolongation of aPTT was observed in 51.9% of our patient population. Patients with aPTT prolongation had statistically higher SAPS3 (p < 0.0001) and SOFA (p = 0.0006) scores, namely 78 and 14 patients as compared to 59.5 and ten patients, respectively.

Proven pathogens were found in 74.7% of our patients, with gram- bacteria being the most common (55.7%). In the course of ICU stay, on the day of the lowest FXII activity, almost half (44.3%) of our study patients developed renal failure and needed renal replacement therapy (RRT). Patients’ baseline characteristics stratified for aPTT are presented in Table 1.

**Fig. 1:**
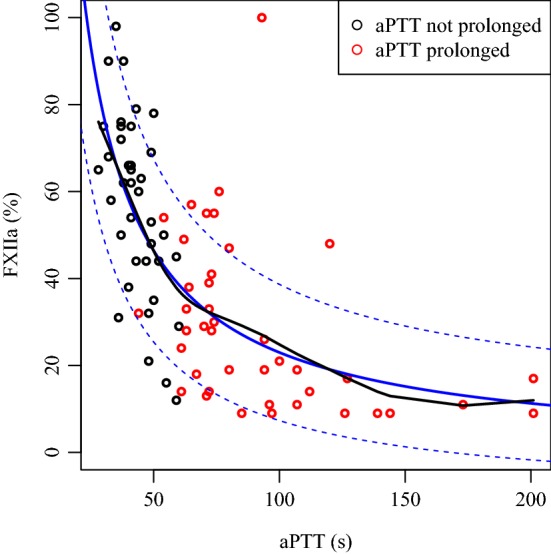
Inverse proportionality of FXII and aPTT measurement. The black line shows the fitted cubic line. The inverse proportionality is depicted as the blue solid line with 95% CIs as dashed lines

In Fig. 1, the fitted cubic smoothing spline (black line) reveals a highly nonlinear dependence between FXII and aPTT levels. Inverse proportionality of FXII and aPTT measurement (blue solid line with 95% CIs as dashed lines) was confirmed by regression analysis (p < 0.0001).

**Fig. 2:**
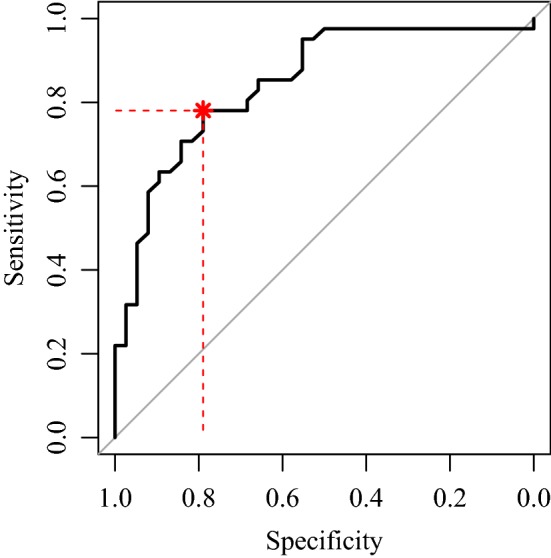
ROC curve for aPTT prolongation predicted by FXII level: AUC 0.85 (0.76–0.93)

The optimal threshold for FXII levels was calculated at 42.5%. Below this, the FXII deficiency is most likely to confound the aPTT assay, resulting in prolongation of aPTT (Fig. 2).

The optimal threshold for FXII is 42.5% and corresponds to the point closest to the top-left marked with an asterisk, where a specificity of 0.79 and a sensitivity of 0.78 are achieved.

Of our patients 50.6% experienced such a FXII deficiency, 80.0% of whom showed prolongation of aPTT. Disease severity and different treatments were associated with a FXII deficiency below the threshold level of 42.5% as presented in Table 2.

**Table 2:** Patient characteristics shown by FXII above and below threshold of 42.5%

Characteristic^a^	Total (n = 79)	FXII ≥ 42.5% (= 39)	FXII < 42.5% (n = 40)	Estimate with 95% CI^b^	p value^c^
aPTT prolonged	41/79 (51.9)	9/39 (23.1)	32/40 (80)	12.77 (4.08–45.2)	< 0.0001
Anticoagulation therapy	51/74 (68.9)	32/36 (88.9)	19/38 (50)	0.13 (0.03–0.47)	0.0004
Septic shock	26/74 (35.1)	6/36 (16.7)	20/38 (52.6)	5.42 (1.7–19.75)	0.0015
Scores					
SAPS3 (pts)	58 (25–74.5)	38 (15–66)	70 (55–82.5)	− 28 (− 44 to − 14)	0.0002
SOFA (pts)	8 (5–15)	7 (4–16)	8 (5.75–13.5)	0 (− 2 to 2)	0.705
Extracorporeal circuit treatments at lowest FXII level					
ECMO	7/79 (8.9)	3/39 (7.7)	4/40 (10)	1.33 (0.21–9.72)	1
RRT	35/79 (44.3)	10/39 (25.6)	25/40 (62.5)	4.73 (1.68–14.25)	0.0014
Total administration of blood products before FXII measurement					
Red blood cell concentrate	56/74 (75.7)	23/36 (63.9)	33/38 (86.8)	3.66 (1.05–15.02)	0.0301
FFP	8/74 (10.8)	2/36 (5.6)	6/38 (15.8)	3.14 (0.51–34.03)	0.2627
Platelet concentrate	25/74 (33.8)	8/36 (22.2)	17/38 (44.7)	2.79 (0.93–9.02)	0.0513
Administration of blood products within 3 days before FXII measurement					
Red blood cell concentrate	25/74 (33.8)	10/36 (27.8)	15/38 (39.5)	1.68 (0.58–5.1)	0.3322
FFP	5/74 (6.8)	1/36 (2.8)	4/38 (10.5)	4.05 (0.38–208.23)	0.3585
Platelet concentrate	15/74 (20.3)	3/36 (8.3)	12/38 (31.6)	4.97 (1.17–30.31)	0.0194
Biological parameters					
Hemoglobin (g/l)	89 (83–94.5)	90 (84.5–95.5)	87 (79–92.5)	3.54 (− 1 to 8)	0.113
Hematocrit (%)	26.5 (24.65–28.4)	26.7 (24.7–28.45)	26.1 (23.95–28.33)	0.7 (− 0.8 to 2.2)	0.3801
aPTT (s)	60 (42–78)	43 (37.5–54)	72.5 (61–101.75)	− 29 (− 40 to − 20)	< 0.0001
PT Quick (%)	59 (43–77)	75 (62–84.5)	45 (32–57)	29 (21–37)	< 0.0001
INR	1.4 (1.2–1.7)	1.2 (1.1–1.3)	1.6 (1.4–2.2)	− 0.4 (− 0.7 to − 0.3)	< 0.0001
Fibrinogen (mg/dl)	376 (228.5–570)	518 (362–656)	250 (201.5–381.5)	221 (127–304)	< 0.0001
Antithrombin (%)	51 (35–66)	64 (56.5–74)	37.5 (19–49.25)	27 (19–35)	< 0.0001
Platelets (G/l)	103 (69–180)	127 (97.5–212.5)	73 (45.5–154.75)	52 (26–84)	0.0009
Leukocytes (G/l)	10.6 (7.4–13.65)	9.7 (7.95–12.7)	11.3 (6.88–14.38)	− 0.6 (− 3.1 to 1.8)	0.6449
C-reactive protein (mg/dl)	10.67 (5.18–15.25)	10.88 (5.21–19.92)	10.37 (5.46–14.3)	0.7 (− 3.63 to 4.81)	0.8322
Procalcitonin (µg/l)	2.46 (0.65–5.8)	0.61 (0.27–2.02)	4.19 (1.93–11.3)	− 2.86 (− 4.76 to − 1.48)	< 0.0001
Thromboembolic event	22/79 (27.8)	7/39 (17.9)	15/40 (37.5)	2.71 (0.88–9.13)	0.0784
Bleeding complication	22/79 (27.8)	8/39 (20.5)	14/40 (35)	2.07 (0.68–6.65)	0.2101

^a^Binary data are presented as no./total no. (%), continuous data as medians (25th–75th percentile)

^b^Estimated odds ratio for binary and median difference for continuous variables

^c^Differences in groups assessed with Fisher’s exact test for binary variables and the Wilcoxon rank sum test for continuous variables

Patients with FXII deficiency were in general sicker than patients without, as evaluated by SAPS3 scores. FXII deficiency was significantly more common in patients with septic shock. Renal replacement therapy was more often necessary as well as the administration of red blood cell and platelet concentrates until three days before FXII measurement. The coagulation tests are deflected; coagulation factors and platelets are decreased in the deficiency group. Except for procalcitonin, which was significantly higher in the deficiency group, there were no differences in the inflammatory parameters. The rate of thrombosis and bleeding complications was not significantly different in either group although the number of thromboses was higher in patients with a FXII deficiency.

**Fig. 3:**
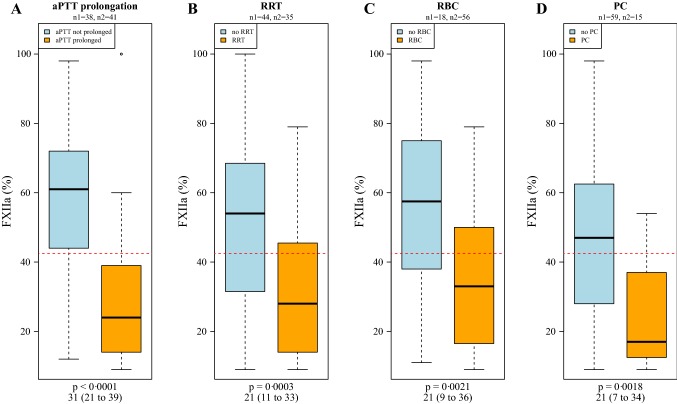
FXII levels depending on aPTT prolongation, RRT and transfusion. FXII levels differ in dependence on aPTT prolongation (**a**) and significant treatments (renal replacement therapy **b**; cumulative red blood cells **c**; platelet concentrates **d**). The red dashed line depicts the calculated 42.5% threshold level of FXII

As depicted in Fig. 3, FXII levels significantly differ in patients with aPTT prolongation as compared to patients without (p < 0.0001), and thus confirmed the calculated FXII threshold level of 42.5%. FXII levels were significantly lower (p = 0.003) when undergoing renal replacement therapy or transfusion of blood products (total RBC: p = 0.0021 and platelet concentrate until three days before FXII measurement: p = 0.0018) was necessary.

## Discussion

Our study showed a significant inverse proportionality between FXII and aPTT. In addition, a threshold FXII of 42.5% could be calculated from this, it comes to a significant extension of aPTT. We would like to point out a careful use and careful interpretation of aPTT for therapeutic decisions.

Correct interpretation of so-called standard laboratory coagulation parameters combined with adequate therapeutic response, especially in the critically ill patient population, may contribute to the reduction in thromboembolic complications as well as to the prevention of bleeding complications. Beside routine measurement of PT/INR and platelet count, adjustment of a patient’s coagulation and thromboembolic prophylaxis and therapy is usually based on aPTT measurement. In this connection we came to the conclusion that spontaneous prolongation of aPTT measurement in critically ill patients is influenced in nearly 50% by decreased Factor XII levels. Therefore, spontaneous prolongation of aPTT is neither able to predict bleeding complications nor helpful in predicting thromboembolic complications, whereas a shortened aPTT may identify patients at risk for thromboembolic complications.

Several confounding factors, such as Lupus anticoagulants or FXII deficiency, prolong aPTT without reflecting the patient’s bleeding tendency or the efficacy of anticoagulant agents. FXII deficiency can be congenital or acquired and differs among different patient populations [20, 21], but exclusively FXII deficiency was shown to be the most common cause of aPTT prolongation in cancer patients [19]. Only a few studies have investigated the effect of Factor XII deficiency on aPTT and to our knowledge no study has to date been conducted in critically ill patients.

This is the first study reporting about the frequency of FXII deficiency in critically ill patients with spontaneous prolongation of FXII and the influence of FXII deficiency per se on prolongation of aPTT in this patient population. In 50.6% of our patients with prolonged aPTT, a relevant FXII deficiency was observed. But we did not detect any significant difference in the occurrence of bleeding complications as compared to those patients without FXII deficiency. A FXII threshold level of 42.5% or less resulted in significant prolongation of aPTT.

In routine practice, spontaneously prolonged aPTT may discourage ICU physicians from initiating thrombosis prophylaxis or therapy. Not only the withholding of antithrombotic drugs, but also the transfusion of fresh frozen plasma in order to normalize the prolonged standard coagulation test is common practice [22, 23].

Our study indicates that FXII deficiency is a common finding among critically ill patients, especially those with sepsis and septic shock. Sepsis severity is associated with elevated procalcitonin levels [24, 25] and, as confirmation thereof, significantly higher procalcitonin levels were also found in our FXII-deficient patients.

As a consequence of infection and inflammation, FXII seems to be consumed by direct activation of FXII by pathogens [26] as well by activation by apoptotic cells via the externalized phosphatidylserines (PS) [27], extracellular nucleic acids such as RNA and DNA [28–30] as happens during neutrophil extracellular trap (NET) formation [31]. Furthermore, activated platelets and the platelet-derived microparticles (PMP) activate FXII via the exposed polyphosphates, such as phosphatidylserine (PS) [32]. Patients with septic shock usually receive norepinephrine, which also amplifies the shedding of such procoagulatory PMPs [33].

Patients undergoing continuous renal replacement therapy (RRT) were seen significantly more often to have a FXII deficiency. This could be explained by FXII activation through contact with artificial surfaces of catheters or in the extracorporeal circuits. Once FXII is activated by a surface, up to 60% of it remains bound to the surface [34], resulting in rapid depletion of FXII in the bloodstream.

FXII deficiency was detected more frequently in patients requiring red blood cell concentrates. Red blood cells are known to shed procoagulant microparticles [35]. However, this finding might also be attributed to the severity of the underlying disease and the related need for transfusion. Nevertheless, not only red blood cell concentrates, but also platelet transfusion may affect consumption of FXII. The significantly lower FXII levels in patients receiving platelet concentrates within three days before measurement indicate that the administration of platelet concentrates may lead to a FXII deficiency. This might happen via activated platelets derived from concentrates, which can expose polyphosphates [32] and subsequently activate FXII resulting in a deficiency. This is emphasized by the fact that platelet activation markers in platelet concentrates increase with storage time [36, 37] and also contain platelet-derived particles [36–38], both of which can activate FXII.

A previous study indicated that low or persistently low serial Factor XII values might be associated with poor prognosis [39]. The reason for this could be the preceding activation of the contact pathway, which might lead to an elevated thrombotic risk and inflammation reaction. Early inhibition of FXII could be a promising anti-thrombotic and anti-inflammatory treatment strategy, especially in sepsis. Furthermore, blocking FXII may also be a suitable alternative for anticoagulation of extracorporeal devices like RRT and ECMO by inhibiting contact activation without increasing the bleeding risk [40–42].

The retrospective design of the study is limiting and precludes assessment of causal relationships. The influences of other factors such as the occurrence of antiphospholipid syndrome, FIX and FXI deficiency or deficiency of plasma kallikrein on the aPTT assay were not assessed and should definitely be considered in a further prospective study. The need to re-calculate the FXII cut-off value for aPTT prolongation after adjustment for deficiencies of these factors is given and the FXII threshold level will probably shift. Furthermore, the presence of disseminated intravascular coagulation (DIC) should also be evaluated, since this can lead to prolonged aPTT. DIC is possible although platelet count and fibrinogen levels in our study are quite high for this disease; at any rate, low FXII levels may indicate DIC [43].

Isolated aPTT prolongation in critically ill patients is rare since aPTT prolongation is most likely due to the sum of pathophysiological processes including liver synthesis.

Nevertheless, awareness about the influence of inhibited FXII on the prolongation of aPTT must be particularly emphasized.

## Conclusion

The effect of FXII deficiency is an underestimated problem in critically ill patients. Correct interpretation of prolonged aPTT remains a challenging problem in critical care medicine.

## Abbreviations

aPTT: Activated partial thromboplastin time
CRP: C-reactive protein
DIC: Disseminated Intravascular Coagulation
FFP: Fresh frozen plasma
FXII: Coagulation factor XII
ICU: Intensive care unit
INR: International normalized ratio
PC: Platelet concentrate
PT: Prothrombin time
RBC: Red blood cells
RRT: Renal replacement therapy
SAPS3: Simplified Acute Physiology Score 3
SOFA: Sequential Organ Failure Assessment
